# The emergence of vampire bat rabies in Uruguay within a historical context

**DOI:** 10.1017/S0950268819000682

**Published:** 2019-04-22

**Authors:** G. Botto Nuñez, D. J. Becker, R. K. Plowright

**Affiliations:** 1 Department of Microbiology and Immunology, Montana State University, Bozeman, MT, USA; 2 Departamento de Métodos Cuantitativos, Facultad de Medicina, Universidad de la República, Montevideo, Uruguay; 3 Programa para la Conservación de los Murciélagos de Uruguay, Museo Nacional de Historia Natural, Montevideo, Uruguay; 4 Center for the Ecology of Infectious Disease, University of Georgia, Athens, GA, USA

**Keywords:** Chiroptera, historical analysis, rabies (animal), spillover, Uruguay

## Abstract

Pathogen spillover from wildlife to humans or domestic animals requires a series of conditions to align with space and time. Comparing these conditions between times and locations where spillover does and does not occur presents opportunities to understand the factors that shape spillover risk. Bovine rabies transmitted by vampire bats was first confirmed in 1911 and has since been detected across the distribution of vampire bats. However, Uruguay is an exception. Uruguay was free of bovine rabies until 2007, despite high-cattle densities, the presence of vampire bats and a strong surveillance system. To explore why Uruguay was free of bovine rabies until recently, we review the historic literature and reconstruct the conditions that would allow rabies invasion into Uruguay. We used available historical records on the abundance of livestock and wildlife, the vampire bat distribution and occurrence of rabies outbreaks, as well as environmental modifications, to propose four alternative hypotheses to explain rabies virus emergence and spillover: bat movement, viral invasion, surveillance failure and environmental changes. While future statistical modelling efforts will be required to disentangle these hypotheses, we here show how a detailed historical analysis can be used to generate testable predictions for the conditions leading to pathogen spillover.

## Introduction

For pathogen spillover to occur, several hierarchical conditions have to be present and aligned [1]. First, an infected reservoir population must be present [2]. In structured populations, demography and behaviour of the reservoir hosts are critical components of pathogen persistence [3]. Alongside persistence of the pathogen, shedding of the pathogen and contacts among reservoir and spillover hosts must overlap in space and time [1]. Finally, the detection of these realised spillover events is itself dependent on the frequency and intensity of spillover as well as and the sensitivity of the surveillance system. Comparing these conditions in times and locations where pathogen spillover does and does not occur presents opportunities to understand the factors that shape spillover risk. In Latin America, after advances in the control of canine rabies, bat-borne rabies continues to threaten human and animal health and the number of reported cases has been increasing in recent years [4, 5]. Although bat-borne rabies has been observed throughout Latin America since the 1900s, this disease is a relatively new phenomenon in Uruguay. As a case study, Uruguay therefore presents a novel introduction of a virus into a monitored and large livestock population.

The first bat-borne paralytic rabies outbreak in livestock was detected in Uruguay in 2007, and the common vampire bat, *Desmodus rotundus* (É. Geoffroy Saint-Hiliare, 1810), was confirmed as the source [6]. In 1 week, 193 cows died from rabies, costing the country around $2 million in immediate vaccination alone [7–9]. Rabies virus isolated from vampire bats or livestock from this first year of outbreaks showed high-genetic similarities but divergence from isolates from southern and northern Brazil [6]; however, due to low sample sizes, data were not sufficient to provide a putative origin for the Uruguay outbreak. No rabies sequences from Uruguay have been published or made available from official veterinary laboratories following this initial assessment. Similarly, limited data are available on vampire bat population structure, with a small number of samples suggesting that vampire bats from northern Uruguay are virtually indistinguishable from those in southern Brazil [10].

The absence of bovine rabies in Uruguay until 2007, and its presence only in the northern region of the country thereafter, likely reflects a change in some of the aforementioned conditions (e.g. reservoir distribution, disease surveillance) to allow the occurrence of the 2007 outbreak and subsequent cases. Ideally, a careful statistical analysis of reservoir host distribution, population density, environmental factors and surveillance systems would facilitate differentiating between the various drivers of rabies virus emergence. However, as described above, much of the quantitative data required for such an analysis is absent in Uruguay. For example, the country lacks data on vampire bat colony size, connectivity and foraging patterns [11], reflecting broader issues with limited research in field mammalogy in Uruguay [12]. In this paper, we therefore present a historical contextualisation for rabies emergence in Uruguay to identify and develop testable hypotheses to differentiate the drivers of emergence. More broadly, we highlight how historical context should be considered as a key component of studying wildlife disease ecology and pathogen spillover.

Uruguay is a special case compared with other Latin American countries affected by vampire bat rabies. Uruguay's predominant landscape is grassland, and forests are restricted to riparian areas [13, 14]. Livestock were introduced during the early 1600s and grew to high densities well before wildlife prey populations were significantly reduced by overhunting. In contrast to most Latin American countries, Uruguay's forest coverage has since increased, although this change is due to an increase in industrial forestry. This trend makes Uruguay very distinctive from both a South American and a global perspective [15, 16]. Furthermore, because livestock-related goods are the main export in Uruguay [17, 18], the cattle population is strictly monitored: 100% of livestock is under herd traceability and over 80% are under individual electronic traceability systems [19]. Herd traceability began in 1827, was codified in 1973–74, and this law was extended to all livestock in 1996 [19]. Therefore, shifts in bovine surveillance are an unlikely explanation for the recent emergence of bat rabies. The expansion of rabies into Uruguay therefore may instead reflect a change in the distribution of the reservoir host or a change in environmental conditions that promote viral transmission, persistence or detectability.

The common vampire bat, *D. rotundus* (É. Geoffroy Saint-Hiliare, 1810), is responsible for most cases of rabies in Latin America [4, 20]. *D. rotundus* and the two other vampire bats (*Diphylla ecaudata* and *Diaemus youngi*) are the only three obligate sanguivorous mammals. *D. rotundus* depends almost exclusively on mammalian blood [21]. As this resource is extensively available, vampire bats have a widespread distribution that may be constrained by temperature because they have poor homoeothermic capacity [21]. Their sanguivorous diet facilitates the transmission of rabies virus through saliva [21–23]. Rabies virus is likely transmitted through frequency-dependent processes such as grooming, blood sharing and aggressive interactions within vampire bat populations. Metapopulation dynamics (specifically, the immigration of infected individuals) may promote viral persistence [24, 25]. Since the introduction of livestock to Latin America, domestic animals are commonly the predominant prey for vampire bats [26, 27]. The intensification of livestock rearing into forested regions or in areas with otherwise small-scale cattle rearing likely drives dramatic dietary shifts, especially combined with defaunation processes (e.g. as in Uruguay). In Mexico, an extrapolation from passive surveillance confirmed an estimated 90 000 to 100 000 rabies-related cattle deaths per year [28]. In Peru, active surveillance corrected for underreporting estimated >400 deaths per 100 000 cattle in 2014 from vampire bat-borne rabies [29].

In some areas of Latin America, increased deforestation and the corresponding reduction of wildlife populations may trigger an increase in vampire bat predation on cattle and increase risks of rabies outbreaks [20, 30–33]. Intensification of cattle production also increases the availability of prey for vampire bats and allows bat populations to increase and disperse [27, 34]. This phenomenon of population increase driven by changes in livestock production is likely dependent on the landscape and the history of each site. For example, in central-southern Brazil, forest fragmentation for grazing areas and croplands has replaced natural wildlife prey with livestock, facilitating vampire bat predation on cattle [35]. In northern Brazil, mining or logging activities in the forest increased contact between humans and vampire bats leading to increased risk for human rabies [20, 30, 32, 36]. In some cropland areas, livestock were removed from residential yards so that humans became the most accessible prey for vampire bats [20, 30]. The increase in rabies in Uruguay has contrasting mechanisms as forest coverage has increased through commercial afforestation and agricultural expansion has led to substitution of natural grasslands.

We propose that the recent emergence of vampire bat-borne rabies in cattle in Uruguay in 2007 could be explained by one or more of the following hypotheses:

- Vampire bats recently extended their range into Uruguay.
- Rabies recently invaded Uruguay, where vampire bats and cattle have been historically distributed.
- Rabies has been recently detected in Uruguay, despite previous circulation in both vampire bats and cattle.
- Vampire bats and rabies have been present in Uruguay, but recent environmental changes have allowed spillover into livestock. These changes have allowed rabies virus to persist in bat populations and cause epidemics in bats that lead to epidemics in cattle.

Given the cattle surveillance in Uruguay, and the accessibility of the entire country, we assume that if an outbreak had occurred, it would have been detected. To assess historical evidence for these alternative hypotheses for viral emergence, we review historical records on: (i) the distribution of vampire bats and the circulation of virus in both (ii) vampire bats and (iii) cattle.

## Recent range expansion of vampire bats into Uruguay

In general, range expansions might be explained by changes in climatic limiting conditions, changes in the distribution of food resources and changes in roost abundance and availability [37, 38]. We collected all historical records of *D. rotundus* in Uruguay, since European colonisation, to examine historical support for the hypothesis that *D. rotundus* has recently expanded its geographical distribution into Uruguay (see supplementary information and Table S1 for a detailed description). We also compiled information available on changes in roost availability and food sources.

### Historical records

**Fig. 1.**
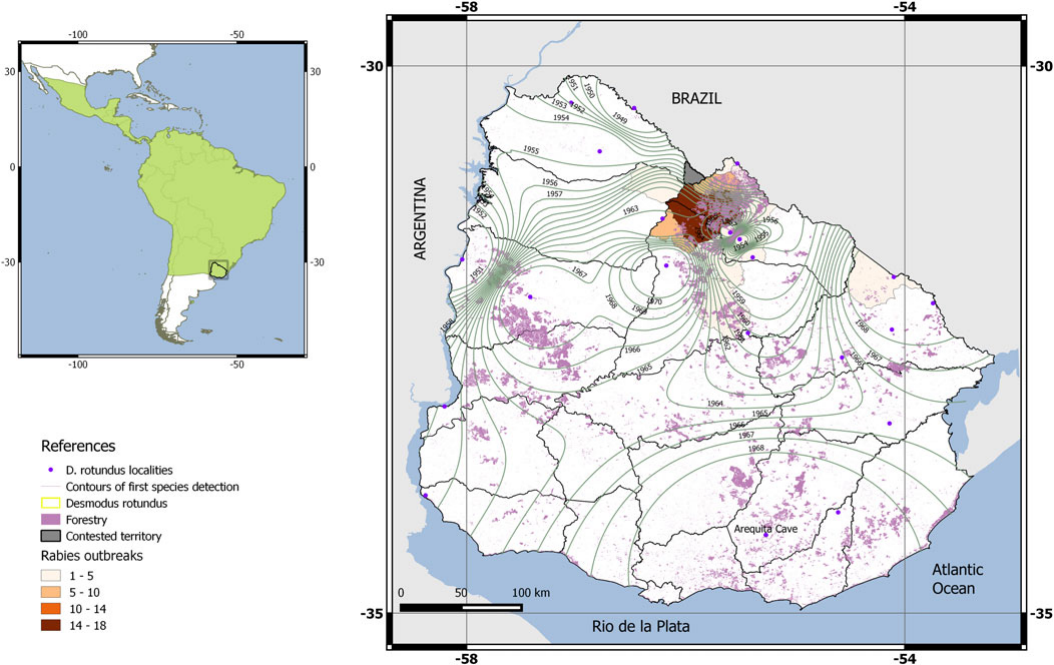
Map showing the continental location of Uruguay, the accepted current distribution of *D. rotundus* (IUCN, 2012), the localities for *D. rotundus* mentioned for Uruguay by Langguth & Achaval (1972), and the contours generated by interpolation of the date of first record of the species in each locality (see supplementary material and Table S1 for detailed discussion). The Arequita cave in southern Uruguay is shown. The cumulative number of ranches affected by rabies outbreaks in Uruguay is presented according to the official information provided by the Uruguay's Ministry of Livestock Agricultures and Fisheries.

The first record of *D. rotundus* in Uruguay was in 1933, but there were reports of cows being attacked by vampire bats for several years beforehand [39]. At that point, *D. rotundus* was considered rare in Uruguay. Less than 40 years after this first record, *D. rotundus* was confirmed in several locations around the country, suggesting a widespread distribution [40–42]. However, this pattern of new records and locations does not necessarily suggest expansion of the vampire bat range but rather an incursion of researchers into formerly unexplored roosts (Fig. 1). There is even a previous description of a cave in south-central Uruguay where a description of bats occupying the site is consistent with *D. rotundus* [43]. While this is still an unconfirmed report, it supports the idea that *D. rotundus* has occupied Uruguay for longer than documented reports or captures suggest. Only one roost in south-central Uruguay may have been recently colonised by *D. rotundus*. The Arequita cave was visited by mammalogists several times between 1890 and 1980, and although a number of bat species were recorded, *D. rotundus* was not found there until 70 years after the first recorded visit [39–42, 44].

### Roosts

Roosts used by *D. rotundus* in Uruguay are mostly caves, abandoned buildings and abandoned mining tunnels [45]. Recent changes in roost availability include short-lived mining activities of the early 1900s and changes in the distribution of rural workers in the late 1900s and early 2000s that may have provided other housing structures [13, 46–49]. However, both processes provide a limited number of new roosts, probably insufficient to explain an expansion of vampire bats. Moreover, while some of the first reports of the species in Uruguay were related to these structures [40, 41], as soon as new areas were explored, the species was detected in many long-available natural roosts. *D. rotundus* is now considered abundant and widespread throughout Uruguay, based on the number of roost registered and the detection of vampire bats by acoustic surveys and mist-netting [11, 45, 50–53].

### Food sources

**Fig. 2.**
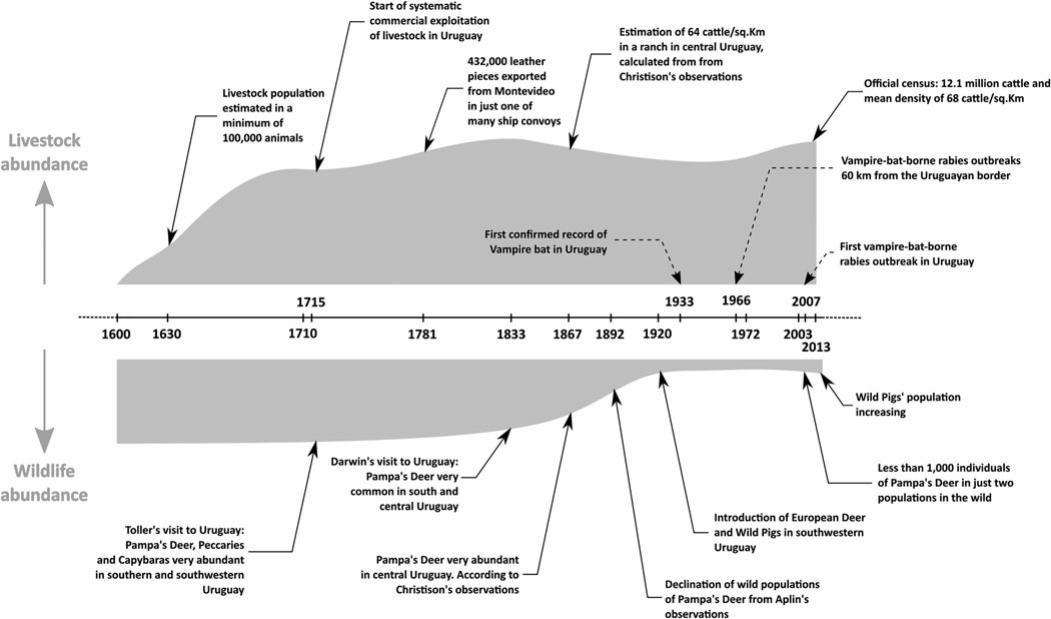
Timeline of livestock and wildlife abundance, vampire bat records and bovine rabies outbreaks in Uruguay. See the text and supplementary material and Table S1 for details.

Livestock were introduced into Uruguay during European colonisation in the late 1500s and early 1600s, mostly through the missionaries from the Company of Jesus [54, 55]. By the 1630s, cattle were abundant in the Uruguayan territory, with estimated minimum 100 000 animals according to Hernandarias [55]. The northern coast of the Rio de la Plata estuary was not occupied by Europeans during most of the 17th century, and livestock were managed free range and not heavily exploited until 1710 [55]. From this point, there is discrepancy among different documentary sources on the number of cattle in the territory of Uruguay (see supplementary material for a detailed discussion and Table S1). However, considering several hundred thousand pieces of leather were exported from Montevideo annually, the cattle population likely was very large [56, 57]. Other direct reports of exports from Montevideo, published notes from travellers, and historic reports from inhabitants, also support the notion of a large cattle population [56, 58]. By 1908, the livestock population was already 8.2 million cattle and 21.5 million sheep as assessed by censuses [59]. According to the last official estimation, the current population is 12.4 million cattle and 7.3 million sheep [60]. The presence of high concentrations of livestock precedes the large declines of wildlife (Fig. 2, Table S1). There is no evidence that climatic conditions or the availability of food and roosts have limited *D. rotundus* populations in Uruguay for the past 100 years.

Uruguay has historically been described as grasslands with forest in the rivers' banks [61], where several wild mammals could have served as prey for *D. rotundus*, including pampa's deer (*Ozotoceros bezoarticus*), marsh deer (*Blastocerus dichotomus*), brocket deer (*Mazama gouazoubira*), peccaries (*Pecari tajacu*) and capybaras (*Hydrochoerus hydrochaeris*) [58, 62–69]. With the exception of capybaras, these wildlife prey species now have low densities, restricted geographic ranges or are locally extinct [45]. This severe defaunation occurred by the end of the 1800s and the early 1900s, when livestock populations were already established. Moreover, during the early 1900s, wild pigs, goats, Asian buffalo and two species of exotic deer were introduced into Uruguay and formed established wild populations [45, 70]. Wild pigs are now widely distributed in Uruguay and could be a food source for *D. rotundus* [70–72]. Axis deer also exist in relatively high densities in the south of the country [70].

### Climate

In relation to climate, more than 40 years ago, McNab proposed that the 10 °C mean minimum isotherm for the coldest month was a key predictor of the geographic range limit of *D. rotundus* [73]. This limit was proposed in accordance with feeding habits of *D. rotundus* and their energetic limitations. Vampire bats are highly sensitive to cold and dehydration owing to their protein-based diet, inadequate lipid stores and high rates of evaporative water loss [73–75]. Cooler climates increase the amount of energy *D. rotundus* must expend to maintain normal temperature, requiring larger bloodmeals. As bloodmeal size is limited by body size and flight capacity, this isotherm restricts the *D. rotundus* distribution [73]. New records of the species after McNab's work have all fallen within his proposed range limit. For instance, records in southern Uruguay, and new records in Mexico and Argentina fall inside the proposed limit [45, 76–78]. Interestingly, the *D. rotundus* distribution does not overlay with cattle distributions in the southern or northern limits (Fig. S1). In Argentina and Mexico, cattle are present on both sides of the 10 °C isotherm, but *D. rotundus* is only present on the side of each isotherm that is closest to the equator. In some areas such as the province of Buenos Aires (Argentina) where *D. rotundus* is absent, cattle densities are higher than in central Argentina where *D. rotundus* is present [79]. Combined, this evidence suggests that the 10 °C isotherm is a good proxy for the *D. rotundus* distribution limit.

While an increase in air temperature has been observed for the region during the 1900s and is expected to continue in the future [80], vampire bats already occupy the entire country. Hence, overall distribution of the species in the country may not be affected. However, behavioural changes (such as feeding habits) might be expected in response to temperature shifts. Increases in minimum temperature and decreases on the frequency of cold nights might impact flight ability of vampire bats, making them able to forage over longer distances [73].

Two recent studies analysed the potential range expansion of vampire bats [81, 82]. One concluded that an extensive expansion into North America is unlikely [81]. Although the other proposed a future range expansion [82], predictions of this species distribution model notably did not include the southernmost area of the known distribution (including Uruguay).

## The possibility of recent rabies introduction into Uruguay

As shown above, the historical record provides no support for the hypotheses that *D. rotundus* recently expanded into Uruguay. Alternative explanations for the recent emergence of bovine rabies in Uruguay are therefore (i) a recent invasion of the virus into Uruguayan bat populations or (ii) a change in conditions leading to increased viral persistence in vampire bat populations and an increased probability of transmission to livestock.

The first report of vampire bat-borne bovine rabies was in 1911, about 700 km from the Uruguayan border in the state of Santa Catarina, southern Brazil [83, 84]. Since this first-reported outbreak, vampire bat-borne rabies in cattle has remained common in the area near the Uruguay–Brazil border [85, 86], suggesting sustained circulation of rabies virus in *D. rotundus*. Even if the late discovery of *D. rotundus* in Uruguay was a reflection of a host expansion process, in 1966 there were already reports of vampire bat-borne rabies in southern Brazil within 60 km of the Uruguayan border [87]. By that time, *D. rotundus* was known to be present in several localities through Uruguay [40, 42]. Rabies in the neighbouring Brazilian southern state of Rio Grande do Sul has been present for at least 60 years. According to one study, between 1964 and 2008, rabies in Rio Grande do Sul has shown a cyclic behaviour with epidemic pulses [88]. Another study, in the same Brazilian state, showed that between 1985 and 2007, only 2 years (1996 and 2001) have had no reported bovine rabies cases in the same state [89]. Accordingly, it is unlikely that rabies virus only recently invaded into Uruguay in 2007. Given the sustained circulation of rabies in southern Brazil, longitudinal seroprevalence in northern Uruguay is needed to understand whether the virus exhibits more sporadic dynamics (perhaps suggesting a more recent invasion) or more endemic dynamics (suggesting longer-established virus) [24, 25].

## Recent detection of circulating rabies

One alternative hypothesis is that rabies has been endemic in Uruguay but was only recently detected through surveillance. However, livestock in Uruguay are subject to robust surveillance, and the small country has no inaccessible or remote areas that are not monitored [19]. Moreover, retrospective studies conducted on samples from cattle that died from undiagnosed neurological disease have tested negative for rabies. In 2011, samples from 193 cattle that died from neurological signs between 1999 and 2011 were tested with direct immunofluorescence, immunohistochemistry and histopathology techniques [90, 91]. Immunohistochemical approaches have proven to be reliable to detect rabies virus in formalin-fixed samples from livestock and wildlife in retrospective studies [92]. No samples were positive for rabies before the 2007 outbreak, suggesting that the absence of notified cases does not reflect a failure in surveillance [90, 91]. The introduction of bovine rabies into Uruguay is therefore likely to be a recent phenomenon. While disease surveillance and livestock tracing system in Uruguay are adequate, publicly available systematic reports on livestock and wildlife testing are needed both from the perspective of surveillance and for the data needed to test the proposed hypotheses.

## Recent environmental changes leading to persistence and spillover

The historical records reviewed above indicate that recent changes in the distribution and abundance of vampire bats or livestock are unlikely to be the main driver of vampire bat rabies emergence in Uruguay. Rabies virus has been circulating in nearby southern Brazil for at least 100 years. The absence of detected bovine rabies cases before 2007 is unlikely to be explained by a failure in disease surveillance, given the robust monitoring of livestock throughout Uruguay.

An alternative hypothesis for the recent spillover of rabies may be a change in pathogen dynamics (e.g. persistence) within vampire bat populations. In general, factors that contribute to pathogen persistence in bat populations include population size, seasonal reproduction, hibernation and connectivity among roosts [93, 94]. Rabies virus transmission is likely to be frequency-dependent in vampire bats, and thus colony size may have little or no effect on rabies transmission [24]; furthermore, the historical records suggest that colony sizes are unlikely to have dramatically changed in the years prior to the outbreak. While culling practices (e.g. use of vampiricides) are associated with increased rabies transmission in vampire bats [24], culling practices only began in response to the 2007 outbreak and thus cannot explain the emergence event. A shift in vampire bat reproduction (e.g. due to seasonality [95, 96]), stemming from climatic factors is also an unlikely driver of rabies emergence, given that there is no evidence for a change in climate seasonality in recent years [97, 98]. Because colony connectivity is critical for explaining patterns of rabies virus persistence within vampire bat populations [24, 25], shifts in vampire bat movement and connectivity could explain the emergence of rabies virus in Uruguay.

The most dramatic environmental change that has taken place in Uruguay recently – an increase in forest coverage – overlapped in space and time with the initial rabies virus outbreaks. This change in forest coverage was observed following the implementation of the Forests Act (Law 15.939, 1988) and peaked during the early 2000s. The change in forest coverage was abrupt, with forest plantations increasing 60.8% from 764 825 to 1 230 013 ha between 2000 and 2011 [99]. All recorded cases of vampire bat rabies have occurred in the area of most intense forestry activity, except for one outbreak in 2014 in the Department of Cerro Largo. Increases in forest coverage and consequent decreases in grassland surfaces were not followed by decreases in livestock numbers. On the contrary, livestock density increased in Uruguay during this same period. The concentration of cattle in small, scattered, dense patches could therefore affect the dispersal of *D. rotundus*, thereby increasing inter-colony connectivity and metapopulation dynamics that facilitate rabies persistence [25]. Vampire bats roosting in a landscape with homogeneously distributed livestock may forage in small distances around the roosts, reducing contact among distant colonies. When the roosting areas are embedded within habitat matrices with patchy distribution of livestock prey, vampire bats may have to travel further to feeding areas that may be already used by other colonies, thus increasing contact among colonies. This is supported by observations that vampire bats preferentially feed on livestock and that their movement behaviour will often track the distribution of livestock [96]. Culling activities could also modify vampire bat movement dynamics and increase rabies transmission within vampire bat colonies [24, 31].

Critical data needs remain to quantify the structure and connectivity of *D. rotundus* in this newly forested region compared with neighbouring regions with and without rabies. Furthermore, two important aspects of *D. rotundus*' biology in Uruguay are absent: population density across the country and predation pressure on livestock. While there are no data on the former, the distribution of livestock – a good proxy of bat population size [24] – does not suggest a higher density of *D. rotundus* in the outbreak area Fig. S2). However, this assumption should be tested by assessing vampire bat densities, feeding activity and roost distribution. Standard acoustic surveys could be used to provide information on bat movement patterns, which can be combined with information on predation pressure of vampire bats on cattle. A recent study showed that vampire bats from the north are almost indistinguishable from southern Brazilian populations [10], suggesting either a southern expansion or a recolonisation of empty roosts after culling activities. More work including southern Uruguayan populations and samples collected before culling activities are necessary to differentiate these hypotheses. Population genetics could also provide data on population size and roost connectivity [100]. Culling has been focused on the eastern half of the country, starting in 2007 in the northeast in response to the outbreak and then extending south. No exhaustive reports of culling activities are available. Systematic assessments of culling are necessary, especially in regard to how they may impact bat dispersal and rabies seroprevalence patterns.

Our survey of the historical record suggests that recent environmental changes that may have modified vampire bat behaviour are a likely driver of rabies virus emergence in Uruguay and that recent host population expansion, viral invasion and improved disease detection are unlikely explanations. Additional analyses can help reject these latter alternative hypotheses. For example, the recent expansion of vampire bats into Uruguay could be tested by assessing the genetic structure of vampire bat populations and the potential effects of culling activities started in 2007 [10, 101, 102]. A genetic analysis of the rabies virus isolates from these outbreaks could also shed light on the previous circulation of the virus in the country; for example, a rabies virus phylogeny was used to show independent invasions of rabies virus into Trinidad from the continent [103]. Rabies virus genetics have also been used to infer the rate of spatial spread in Peru [31]. However, limited rabies virus isolates currently constrain these analyses for Uruguay. Accordingly, further work on viral detection and isolation in vampire bats should be conducted in Uruguay. Last, and most important, the primary hypotheses of environmental change must next be tested with available spatial and temporal data on bat population size and distribution, forest cover, livestock density and rabies outbreaks. Because such analyses will be limited by data scarcity, new data collection efforts are needed to assess this hypothesis.

## Conclusion

Given this historical context of vampire bat and cattle distribution in Uruguay, a likely explanation for the recent emergence of vampire bat rabies in Uruguay is the substitution of native grasslands with forest plantations that could have altered vampire bat movement and promoted viral persistence, leading to increased transmission from *D. rotundus* to cattle. Spatial analyses of landscape structure between northern Uruguay (where rabies persists) and neighbouring areas where rabies does not persist could help test this hypothesis. Spatial analyses of epidemiological data could be complimented by field surveys of the population structure, connectivity and feeding behaviour of *D. rotundus* in these same areas. More broadly, our case study on bovine rabies emergence in Uruguay provides an example of how a detailed historical analysis on reservoir host distribution, ecology and disease occurrence can help develop and evaluate alternative hypotheses for understanding the determinants of pathogen spillover. Even when basic conditions for spillover appear to be present, analyses of historical contexts and local landscape characteristics can provide testable hypotheses about pathogen emergence and persistence and should be considered more generally when studying wildlife disease ecology.

## Supplementary material

For supplementary material accompanying this paper visit https://doi.org/10.1017/S0950268819000682. click here to view supplementary material
