# Supplementary material for: The emergence of vampire bat rabies in Uruguay within a historical context
Source: Epidemiol Infect. 2019 Apr 22;147:e180. doi: 10.1017/S0950268819000682 (PMC6518465; doi:10.1017/S0950268819000682)
Supplement: Supplementary file 1 [file S0950268819000682sup001.zip › S0950268819000682sup001/Supplementary Material_Hist_revision_accepted_changes.docx]

**Epidemiology and Infection**

**The emergence of vampire bat rabies in Uruguay within a historical context**

Germán Botto Nuñez, Daniel J. Becker, & Raina K. Plowright

**Supplementary Material**

HISTORY OF THE DISCOVERY OF *D. rotundus* IN URUGUAY AND THE SOUTHERN CONE OF SOUTH AMERICA AND URUGUAY

*Desmodus rotundus* was originally described by E. Geoffroy Saint-Hiliare in 1810 [1]. However, this species was mentioned earlier in several reports of naturalists visiting South America. In his reports of travel along the Amazonas river in 1743, Charles Marie La Condamine described bats that suck the blood from cows, horses, and even humans as common in warm American countries [2]. According to La Condamine’s report, these attacks had been described since the early missions in the Amazonas basin during the 1600s. La Condamine even attributed the massive mortality of livestock introduced in Borja (currently in Loreto Department, Perú [4.44° S, 77.59° W]) and in other missions to bites from *D. rotundus* [2]. Those reports were also mentioned by Buffon, but he described *D. rotundus* without direct contact with this species, based only on other’s descriptions [3]. In his description of *D. rotundus* (“El Mordedor” [The Bitter] in his non-linnean nomenclature), Felix de Azara reject the report of La Condamine as overstated, arguing that the loss of blood is minimum and that there is no other significant damage produced by *D. rotundus*’ bite [4]. Azara himself was bitten by a *D. rotundus,* but he described the bite as a minor wound, claiming that it would be impossible for a livestock herd to be killed by bites from *D. rotundus* [4]. When Azara described *D. rotundus*, he didn’t provide the putative distribution for the species [4]. In 1959, Angel Cabrera in restricted the type locality to Asunción (25.30° S, 53.64° W) *sensu* [5], based on the diaries and notes from Azara.

Charles Darwin was the first naturalist after Azara to actually see a *D. rotundus* feeding. He described the feeding behavior in his diary, and that passage is included by Waterhouse in the description of the species [6]. Darwin recorded the species in the central-northern region of Chile (Coquimbo, IV Region; 29.99° S, 71.35° W). He proposed that *D. rotundus* should be absent or unknown in central Chile, citing a report from Molina, who dismissed the presence of “blood-sucking species” in the central region [6], suggesting that the species was restricted to northern Chile only.

Another early record of *D. rotundus* comes from Alcide D’Orbigny, who collected some individuals in Bolivia (San José de Chiquitos, north of Department of Santa Cruz, 17.84° S, 60.74° W) during his travel around South America [7]. Interestingly, even though he travelled throughout the continent, the only mention of the species was from Bolivia [7]. Hermann Burmeister included *D. rotundus* in his “Description Physique de la Republiqué Argentine”, noting that the species was probably present in Argentina but restricted to the northern border, given records of the species in southern Paraguay [8]. However, his notes are restricted to individuals collected in Lagoa Santa in Brazil [8] (Minas Gerais, 19.63° S, 43.90° W).

In 1894 J. H. Figueira published a list of the 55 known species of mammals from Uruguay [9,10]. Figueira mentioned five bat species comprising the three families currently recognized in Uruguay (Phyllostomidae, Molossidae, and Vespertilionidae). He considered all five species widely distributed along the country, but he mentioned *Plecotus velatus* (*Histiotus velatus*) as the most common, and especially mentioned the Arequita Cave (Department of Lavalleja, 34.28° S, 56.26° W) as one of the known roosts [9]. These data are especially interesting as *H. velatus* is an easily distinguishable species due the size and form of the ears. Currently, *H. velatus* do not inhabit that cave; the cave now has colonies of *D. rotundus* and *Myotis* species [11].

In 1929, Collin Sanborn published “The Land Mammals of Uruguay”, reporting the results of the Captain Marshall Field Brazilian Expedition during the four-month stay in Uruguay [12]. Sanborn visited the Arequita Cave by the end of December 1926 and collected 43 *Myotis chiloensis ater*. These were later determined to be *M. levis* by Alfredo Ximenez following the identification by Richard La Val on specimens from the National Museum of Natural History in Montevideo [13]. No *Histiotus* were recorded here, so Sanborn argued that it was impossible to know if Figueira misidentified the bats or if the colony of *Histiotus* was expelled and replaced by *Myotis* [12]. The currently accepted idea is that the first identification by Figueira was mistaken. In either case, neither Sanborn nor Figueira made any mention of *D. rotundus* in the cave.

In 1935, the first reference to *D. rotundus* in Uruguay appeared in Garibaldi Devincenzi’s work on the mammals of Uruguay [14]. The author referenced ongoing reports of cattle attacked in the north of the country. He instructed several collectors to look for bats in Rivera Department, and specifically for *D. rotundus*, which could be easily differentiated from other bat species from Uruguay. In 1933, one of the collectors submitted a male *D. rotundus* from Centurión in the Department of Cerro Largo (32.14° S, 53.78° W) in the North-eastern region of the country [14,15]. Since this first record, and until the publication of the 1935 paper, no other individual was obtained, so Devinzenci proposed the species to be rare for Uruguay [14]. For some years, the species was supposed to be low in abundance and restricted to the area where it was first recorded. However, between the first record in 1933 and 1960, *D. rotundus* was confirmed in several localities of the country, suggesting a widespread distribution [16]. It is interesting to note that both Devincenzi and Eduardo Acosta y Lara also visited the Arequita Cave before 1950 and again recorded the presence of *Myotis levis*, but did not mention *D. rotundus* [14,15]. The first record of *D. rotundus* inhabiting Arequita Cave is from 1969, mentioning a small number of individuals [17]. These authors also provide several new localities confirming the widespread distribution of *D. rotundus* in Uruguay [17]. In their 1959 paper, Acosta y Lara described *D. rotundus* in a cave in the south of Uruguay (Salamanca Cave, Department of Maldonado, 34.08° S, 54.61° W), noting that there were also *M. levis* present; however, these were restricted to small fissures in the rock where a knife blade could be barely introduced [16]. The authors speculated that *Myotis* may have been forced into these fissures by the introduction of *D. rotundus* to the cave [16]. In 1972 Alfredo Langguth and Federico Achaval published the only paper on *D. rotundus* ecology in the country, based on a colony from Rivera department [17]. In that paper the authors also provide a list of 21 localities from where *D. rotundus* had been collected [17].

Currently, the colony of *D. rotundus* in Salamanca Cave is still present, as are *Myotis* (Pers. Obs. GBN). However, in Arequita Cave, a colony of *D. rotundus* and a colony of *Myotis* co-inhabit the same cave but at intervals. The *Myotis* are present in the cave during the spring and the summer; when the *Myotis* colony increases its numbers in mid spring due to immigration the colony of *D. rotundus* is displaced from the main cave to the atrium of the cave (Pers. Obs. GBN). This behavioral pattern was observed by one of the authors (GBN) and confirmed by the owner of the property, who has systematically observed the cave over many years.

After the work of Acosta y Lara, and Langguth and Achaval [15–17], *D. rotundus* has been registered in many other localities, including localities in the Atlantic cost of the country (Pers. Obs. GBN) and is presumed to be present in the whole country including the rural outskirts of Montevideo city [18]**.** Roosts used by this species in Uruguay are mainly caves, mines, and abandoned buildings (ranging from houses to industrial settlements); the size of the colonies ranges from a few individuals to several hundred or even thousands [18].

Since the first paralytic rabies outbreak in Uruguay in 2007, several campaigns have been conducted by the animal health authorities to reduce *D. rotundus* populations [19–21]. The main procedure for colony reduction is poisoning using Vaseline-based Warfarin pomade (i.e., vampiricide) [19–21]. While the numbers of *D. rotundus* killed in these campaigns remains unclear, several hundred individuals have been collected for diagnostic and surveillance purposes during the first years after the rabies outbreak. The reduction of colonies continues to be one of the main strategies to control the spread of rabies without considering the potential effect of this practice on the circulation of the virus in the bat population [22].

LIVESTOCK INTRODUCTION AND CURRENT ABUNDANCE

Common knowledge in Uruguay states that livestock was introduced in this territory by Hernando Arias de Saavedra (a.k.a. Hernandarias) in the early 1600s [23,24]. However, the introduction by Hernandarias was probably of little impact, because of the relatively small number of livestock; however, the introduction of livestock from the Missionaires from the Company of Jesus was much more important, in number of individuals [23]. The missionary livestock seems to have had several introduction events. First, there was a report of around ten cows bought from the Portuguese ca. 1552 and herded to Paraguay [23,25]. Second, another group of livestock (“several hundred”) was brought from Santa Cruz de la Sierra (Bolivia, 17.78° S, 63.18° W) in 1568 [25]. There were two other introductions from Coquimbo (Chile, 29.99° S, 71.35° W) and Santa Cruz de la Sierra around 1557, to Cordoba (Argentina, 31.41° S, 64.19° W) and Santiago del Estero (Argentina, 27.78° S, 64.26° W). These two stocks were used in the foundation of Santa Fe (Argentina, 31.64° S, 60.71° W) in 1573 [25]. The two accepted origins for livestock in Uruguay are the introduction from the Jessuitic Missionares in the north, forming the “Vaquería del Mar” [23] and the two introductions by Hernandarias in southwestern Uruguay [23,25]. The first introduction by Hernandarias was based in the confluence of the Negro and Uruguay rivers in 1611 (33.28° S, 58.40° W). The second introduction was based in the coast of what is now Colonia del Sacramento (Department of Colonia, 34.47° S, 57.84° W) in 1617 [24]. Both the introductions of Hernandarias’ consisted of a small number of livestock.

By 1627, Hernandarias estimated the population of livestock in the Banda Oriental (current Uruguay) to be about 100,000 animals [25]. This vision of abundant livestock in the Uruguayan territory by the 1630s is shared by many researchers [23]. The Uruguayan territory was not occupied by the Europeans during most of the 17^th^ century, so livestock were not heavily exploited until 1710 [25]. The cattle were first raised as free ranging in both banks of the Rio de la Plata [25].

There are few references about the expansion of livestock after this introduction. However, from the reports of early visitors of Uruguay and from scattered references about leather commerce, one can get an idea of the abundance of livestock. In 1715, William Toller arrived Uruguay in his voyage to Buenos Aires from England travelling aboard the Warwick [26,27]. The Warwick’s first stop in Uruguay was in Castillos Bay (34.34° S, 53.78° W), in what today is the Department of Rocha, during June 1715 [26,27]. Toller and Dover went ashore several times hunting 10 bulls. Toller noted in his diary that “the plains were full of cattle, but most of them Bulls” [27]. He also described many small deer. During the next stops in the north shore of the Rio de la Plata, Toller mentioned the abundance of cattle. He also mentioned deer and 400 peccaries near the St. Thomas river (today the Rosario river, Department of Colonia, 34.43° S; 57.34° W) [27]. The first observation was about 400 kilometers from where Hernandarias introduced livestock some 100 years earlier. Toller repeatedly refers to the poor soil in the places where he recorded high numbers of livestock. Leather commerce was one of the biggest economic activities during the first years of the Spanish colonies in the Rio de la Plata. According to the “Asiento de Negros”, the official Spanish Crown’s record of the slave commerce, between 1702 and 1714, approximately 174,000 leather pieces were exported from Buenos Aires (Montevideo was not founded yet) to England [26]. The British South Sea Co., which had an office at Las Vacas stream (Department of Colonia, 34.00° S, 58.29° W), exported around 480,000 leather pieces between 1723 and 1729 [26]. In 1777, 364,534 leather pieces were exported to Spain from Montevideo and these do not account for smuggling or the slave trade [28]. According to a letter from Perez Castellanos dated in 1787, 321,450 leather pieces where exported from Montevideo in the same year; several came from Buenos Aires, but most were from Uruguay [29]. According to the same author, on March 5^th^, 1781, a convoy departed from Montevideo carrying 432,000 leather pieces; at the same time, six mail frigates and other ships also carrying leather departed to Europe [29]. Around the same date, in 1780, a livestock census showed 408,000 cattle in just Montevideo’s jurisdiction alone [28]. Between 1792 and 1793, 1.6 million leather pieces were exported from Montevideo to Spain [28]. The livestock were raised free-range and with little or no care and were usually killed to sell the leather. During the end of 1800s, ranchers began to castrate bulls in response to the decreasing prices of leather overseas [29]. By 1800, leather exports were stopped due to wars within the viceroyalty. This almost completely stopped cattle slaughtering in the country [28]. In 1806, John Mawe, an English geologist visited Uruguay and was confined to imprisonment in a ranch in central-south Uruguay, in a locality called Barriga Negra (33.97° S, 55.07° W), about 145 km northeast from Montevideo [30]. After his observations of the area, Mawe mentioned the presence of several livestock ranches with herds ranging from 60,000 to 200,000 cattle each [30]. According to census data, there were 2.6 million cattle in 1821, just in possession of Brazilian subjects and in the southern half of the country. By 1887, Brazilians were estimated to possess about 5.2 million cattle (half of that in the south and half in the north) and this would represent less than a half of the estimated 12 million total cattle population in the country [28].

In 1880, David Christison, an English naturalist published the diary of an excursion to central Uruguay, which took place in 1867 [31]. Christison described the country as a continuous grassland with very soft slopes that were rarely interrupted by linear forests associated with some of the rivers. He travelled from Montevideo to San Jorge (Department of Durazno, 32.84° S, 55.89° W) during the autumn. In this district, limited by the Negro river, and the Chileno and Carpintería streams; 60,000 cattle, 100,000 sheep, and 6,000 horses were kept at that time (1880) [31]. Christison estimated the area of the district as 943 km^2^ [31] (which is an accurate estimation when compared to the area calculated from modern maps) producing an average density of almost 64 cattle per km^2^. While it could be supposed that this density is higher than the national average, Christison’s mentioned the abundance of cattle throughout the entire trip, suggesting the density observed in San Jorge was similar to elsewhere in Uruguay. Christison also referenced the recent implementation of wire fencing, not only in San Jorge but also in other areas of the country [31]. Wire fencing was an important advance towards livestock production in Uruguay, improving the management of the herds. During the 1900s, Uruguay began a livestock census that covered the whole country and standardized data collection. In 1908, the agricultural census recorded 8.2 million cattle and 21.5 million sheep [32]. The estimated cattle population was around eight million until the second world war, when a decrease in cattle abundance was observed: 6.3 million in 1943 and 6.8 million in 1946 [32]. While the cattle population in the 1900s was lower than estimates from the previous century, there is an important contribution of sheep that, despite having more fluctuating numbers, averaged more than 17 million during the period [32] (Table S1).

While *D. rotundus* was first recorded in Uruguay during the first half of the 20^th^ century, ample prey was available in great numbers for at least 200 years prior. In 2016, the total estimated livestock population was 12.1 million cattle [33], while in 2011 (the last agricultural census) the population was 10.7 million cattle, 7.3 million sheep, 354 thousands horses, and almost 184 thousands pigs [34]. The density of cattle for 2016 is estimated to be approximately 68 cattle/km^2^, slightly above the estimated density for San Jorge in 1867 [31]. In 2016, 2.23 million cattle were slaughtered for domestic consumption or export and 225,000 were exported alive, totalizing almost 2.5 million animals removed annually from Uruguay [33].

NATIVE AND INTRODUCED WILDLIFE IN URUGUAY IN RELATION TO *Desmodus rotundus* POPULATIONS

.

In 1715, William Toller arrived to the Rio de la Plata on an English cargo ship from an English trade company on route to Buenos Aires [26]. The diaries of both Teller and the president of company responsible for the cargo ship (Thomas Dover) provide data about the area 100 years after the introduction of livestock [26]. Their first stop in Uruguayan territory was at Castillos’ Bay, in what now is the Department of Rocha (34.34° S, 53.78° W) in June, 1715 [26,27]. At this stop, Toller reported the presence of many small deer, probably referring to pampa’s deer (*Ozotocerus bezoarticus*) [27]. He mentioned that some members of the expedition reported to have seen bears; this is likely impossible because of X. Raúl Vaz Ferreira, noted that capybaras (*Hydrochaerus hydrochaeris*) may have looked like bears to the English observers [35]. During that expedition and the subsequent stops in Uruguayan territory, Toller continued to describe wildlife, describing a herd of 400 peccaries after passing Montevideo bay, which he named the St. Thomas river [27]. According to Vaz Ferreira, the St. Thomas river might be the Rosario river (in the Department of Colonia) or the Cufré stream (in the border between Departments of Colonia and San José) [35]. This site is likely the Rosario river (34.43° S; 57.34° W) in the Department of Colonia, judging from the maps on the original manuscript from Toller. There are three recognized species of peccaries in South America: *Catagonus wagneri* (chacoan peccary), *Pecari tajacu* (collared peccary) and *Tyassu pecari* (white-lipped peccary) [36]. The chacoan peccary is restricted to the Gran Chaco in Paraguay and northern Argentina and moves in solitary or small herds of up to ten animals [37]. The other two species are known to inhabit forests; however, according to Felix de Azara’s observations, in the absence of forests, *T. pecari* forms large groups and inhabits “pajonales” (grasslands dominated by *Paspalum*, *Panicum* or *Cortadeira spp.*) [38]. *T. pecari* is known to have larger herds than *P. tajacu*, with groups up t hundreds and with anecdotal reports of up to 2000 individuals [39–41]. Vaz Ferreira assigned the observations from Toller to *P. tajacu* [35], which was supposed to inhabit northern Uruguay, even though it is now considered extirpated in Uruguay [18]. Toller also reported deer (probably the same species than in Castillos: *O. bezoarticus*) in the mouth of the Santa Lucia river and in the Department of San Jose [27]. Cattle were referenced constantly during all the trip, and Mr. Dover hunted cattle several times [26,27]. From the wild species observed by Toller, both deer and peccary could have been feasible food sources for *D. rotundus*. In 1806, John Mawe also provided a description of peccaries in central-south Uruguay also describing “considerable herds of small deer” [30]. Given the mention of large herds, Mawe was probably referring to pampa’s deer.

For other potential prey of *D. rotundus*, Azara [38] suggested that tapir (*Tapirus terrestris*) could have occupied the Rio de la Plata, but likely in low abundances. There are no other mentions to this species for the Uruguayan territory, and from Azara’s notes, it does not seem to have been observed that far south [38]. For deer, Azara confirmed the presence of just one species for the Rio de la Plata basin: the Güazú-ti or pampa’s deer (*O. bezoarticus*). Other species that could have been present in the territory alongside those included in Azara’s list are the marsh deer (güazu-pucú; *Blastozerus dichotomus*) and the grey brocket deer (guazú-tí; *Mazama gouazubira*); for the latter, Azara was not aware of this species occurring in Uruguay [38]. The marsh deer is thought to have been present in Uruguay territory, although is now exitirpated, and the grey brocket deer is still present [18]. The grey brocket deer is a small deer, most commonly observed in forests, which could make it less conspicuous than the pampa’s deer. Because Azara only used local names (mostly in the Guaraní language), we relied on the interpretation of Mones & Klappenbach to assign Latin binomial names[42].

Charles Darwin visited the Rio de la Plata during the Beagle’s trip in 1833. From his observations [43][44], we can extract a good description of the Uruguayan territory in the 19^th^ century. Darwin described Uruguay as an “ondulating surface, clothed with turf” [44]. During his stay in Maldonado (southern Uruguay, 34.90° S, 54.95° W), Darwin described the pampa’s as the only highly abundant mammal species [43], present in all the Rio de la Plata’s coast. Another highly abundant species was the capybara. Darwin attributed its high abundance to infrequent hunting due to its low-value fur and unattractive meat [43].

Several species of native mammals were also mentioned in Christison’s travel report,. Christison mentioned pampa’s deer as “not uncommon” all around the country. He also mentioned a larger deer, perhaps referring to *B. dichotomus*, as previously abundant in the area but then absent for several years. The same comment was made about the jaguar (*Panthera onca*) and the great ant eater (no species mentioned, but could refer to *Mymercophaga tridactyla*), while mountain lions (*Puma concolor*) were seem to be rare but still present [31]. During 1892 and 1893, another English naturalist, Oliver Aplin, visited Uruguay and published on the mammals of the country, mainly based on his observations in the Departments of Rio Negro and Soriano [45]. From the list of mammals presented in this work, deer would have been the most important food source for *D. rotundus*. Aplin mentioned that the pampa’s deer was almost extirpated from the Departments of Rio Negro, with only one herd of approximately 20 animals remaining in the area of Merinos (Department of Rio Negro, 32.38° S, 56.90° W) but still abundant in the Department of Florida [45]. For marsh deer, Alpin described this species as rare but still present in the forests along the Uruguay river’s banks [45]. It is interesting to note that the same year of Aplin’s publication, Figueira also published a paper on the mammals from Uruguay. Figueira considered the pampa’s deer to be common throughout the country but especially abundant in Department of Rocha, while he described the marsh deer as present only the north (maybe coincident with Aplin’s observations) and southeast of Uruguay [9]. Figueira also considered the grey brocket deer to be rare and only present in Departments of the north and southeast [9]. Figueira also mentioned the collared peccary as almost extinct in Uruguay and only present in the northern region in the Departmetns of Artigas and Cerro Largo [9]. Sanborn visited Uruguay 33 years later and described the pampa’s deer as very abundant only in the Department of Rocha, while in low abundances elsewhere [12]. About the marsh deer, Sanborn was told that could still be present in the same area, but in very low abundances [12]. Sanborn did not provide new information on the grey brocket deer, as his comments about this species were only based in Figueira’s and Aplin’s publications. Today, the pampa’s deer is currently limited to two very restricted populations representing two distinct subspecies: *O. b. uruguayensis* in Los Ajos (Department of Rocha, 33.58° S, 54.04° W) and *O. b. arerunguaensis* in El Tapado (Department of Salto, 31.81° S, 56.66° W). These two populations have restricted areas and small population abundance, about 300 individuals in Los Ajos and around 500 in El Tapado [46]. The grey brocket deer is probably present throughout Uruguay, except for the southwest, but not in high densities, and it has been effectively recorded in 10 out of the 19 departments [18,46–48]. As this species uses native and mixed forests as its main habitat, the increase of forestry may have helped it. The marsh deer is considered extinct in Uruguay [18,46], as the last reported record for the species is from 1958 [46].

From these historical reports, we can deduce that the deer populations had declined by the end of the 19^th^ century and the early 20^th^ century. Other native wildlife that could be a food source for *D. rotundus*, such as other deer species, are either extinct (marsh deer) or exist in low abundances (grey brocket deer). Peccary, once possibly abundant, according to the Toller’s and Azara’s reports, were absent from historical records or were considered to exist in very low numbers by the end of 19^th^ century; peccaries are currently considered extirpated [18]. The lesser anteater (*Tamandua tetradactyla*) was mentioned by Figueira as rare and restricted to the north and northeastern regions [9]; similarly, *T. tetradactyla* is now considered rare and restricted to the low mountain ranges in northwestern Uruguay [18]. The giant anteater (*M. trydactyla*) is also considered extirpateds [18]. The jaguar (*P. onca*) is now considered extirpated, and the mountain lion (*P. concolor*) is rare, with confirmed records only in the northern half of the country since 1970 [18].

Other native mammals known to inhabit Uruguay are probably not a good food source *D. rotundus*, either for their small size and/or because of their behavior. There is one report of *D. rotundus* preying on capybaras (*H. hydrochaeris*) in Argentina [49], and the capybara has been common throughout Uruguay since colonial times [18,27,35,43]. .

Other than livestock, there are some introduced naturalized exotic mammals in Uruguay that could represent a food source for *D. rotundus*: two exotic deer (*Axis axis* and *Dama* *dama*), wild pigs (*Sus scrofa*), goats (*Capra hircus*), and Asian buffalos (*Bubalus bubalis*) [18,50]. The Axis deer (*A. axis*) was introduced in the early 20^th^ century (ca. 1920) in south-western Uruguay (Anchorena’s Ranch, Department of Colonia, 34.28° S, 57.97° W), and later one population was translocated to the south-eastern region (Department of Rocha) [50]. Today, this species is distributed almost throughout the whole country but more densely in the Uruguay river basin and in the southern region (Departments of Florida, Canelones, Rocha and Treinta y Tres) [50]. The European deer (Gamo, *D. dama*) was introduced into southern Uruguay in the Department of Florida and has a small population that has shown little or no dispersion [50]. There are some populations of naturalized goats (*C. hircus*) in the Haedo’s and Cuchilla Grande low mountain ranges in the departments of Lavalleja and Maldonado [50]. By the 1980s, two populations of Asian water buffalo were introduced in northern Uruguay: one in Artigas and the other in Rivera departments [18,50]. In Rivera, the population has been restricted to forestry production areas and in Artigas is located in grasslands in the Uruguay River’s basin. The animals in Artigas have been individually identified and monitored using the same system as livestock, as part of the system to control foot and mouth disease’s control in the early 2000s. Finally, wild boar (*S. scrofa*) are now distributed across the country, occupying mainly forests and wetlands [18,50]. This species was introduced in 1920 in the Department of Colonia (Anchorena’s ranch, 34.28° S, 57.97° W) for hunting. Today, wild boar are considered to be agricultural pests, and hunting is permitted and encouraged by the government as a population control measure [50,51]. Studies from southern Brazil demonstrate that *D. rotundus* will feed on *S. scrofa*, especially in forested areas with low livestock density [52,53]. The population abundance estimate of wild boars is not known, but is considered to be currently increasing and widespread in Uruguay [50]. The population size of other wild prey is negligible at a national level, while some species could have some importance for *D. rotundus* at a very local scale..

ON THE GEOGRAPHIC EXPANSION OF *Desmodus rotundus*

Recently, two studies have focused on the potential expansion of distribution range of vampire bats, in response to climate change, and its relationship with increased bovine rabies risk [54,55]. The first study proposed that the distribution of *D. rotundus* would expand under three of the evaluated climate change scenarios [55]. The second study focused in the potential future expansion into the US, and found that while there are some suitable areas in the Mexico-US border, an extensive expansion is unlikely [54]. The analysis performed in the first paper, however, presents some limitations. In reference to the present work, the distribution model used by these authors fails to predict the current southern limit of the species’ distribution [5,55–57]. This limitation constrains the interpretability of the paper’s result for the southern area of vampire bat’s distribution.

SUPPLEMENTARY REFERENCES

1. **Geoffroy Saint-Hilaire E**. Sur les Phyllostomes et les Megadermes, deux genres de la famille des Chauve-souris. *Annales du Muséum National d’histoire Naturelle* 1810; **15**: 157–198.

2. **La Condamine C-M de**. *Relation abrégée d’un voyage fait dans l’interieur de l’Amérique méridionale*. Paris: Académie des sciences, 1745.

3. **Buffon GLLC de**. *La Roussette, la Rougette et le Vampire*. *Histoire naturelle, générale et particuliére, avec la description du Cabinet du Roi. Tome Dixième*. Paris: L’Imprimiere Royale, 1763, p. 55–65.

4. **Azara F de**. *Apuntamientos para la historia natural de los quadrúpedos del Paragüay y Rio de la Plata. Tomo segundo*. Madrid: Imprenta de la Viuda de Ibarra, 1802.

5. **Barquez RM, Mares MM, Brown JK**. The Bats of Argentina. *Special Publications. Museum of Texas Tech University* 1999; **42**: 1–275.

6. **Waterhouse GR**. *Mammalia*. In: Darwin C, ed. *The zoology of the voyage of H.M.S. Beagle, under the command of Captain Fitzroy R.N. during the years 1832 to 1836*. London: Smith, Elder and Co., 1839, p. 1–258.

7. **D’Orbigny AD**. *Voyage dans l’Amérique méridionale: le Brésil, la république orientale de l’Uruguay, la République argentine, la Patagonie, la république du Chili, la république de Bolivia, la république du Pérou. Tome IV. 2 Parte. Mammiféres*. Paris: Pitois-Levrault et ce., 1847.

8. **Burmeister H**. *Description physique de la République Argentine. Tome 3: Animaux Vertébrés. 1 Parte: Mammiféres vivants et éteints*. Buenos Aires: Impimerie de Paul-Émile Coni, 1879.

9. **Figueira JH**. Enumeración de mamíferos. Contribución al conocimiento de la fauna uruguaya. *Anales del Museo Nacional de Historia Natural de Montevideo* 1894; **2**: 187–217.

10. **González EM, Lessa EP**. *Historia de la mastozoología en Uruguay*. In: Ortega J, Martínez JL, Tirira DG, eds. *Historia de la mastozología en Latinoamérica, las Guyanas y el Caribe*. Quito y México DF: Editorial Murciélago Blanco y Asociación Ecuatoriana de Mastozoología, 2014, p. 381–404.

11. **Botto Nuñez G**. *Propuesta de Sitio de Importancia para la Conservación de los Murciélagos (SICOM), Gruta Arequita (Dpto. Lavalleja, Uruguay)*. Montevideo, 2017.

12. **Sanborn CC**. The land mammals of Uruguay. *Field Museum of Natural History Zoological Series* 1929; **17**: 147–165.

13. **Ximénez A, Langguth A, Praderi R**. Lista sistemática de los mamíferos del Uruguay. *Anales del Museo Nacional de Historia Natural de Montevideo* 1972; **7**: 1–49.

14. **Devincenzi GJ**. Mamíferos del Uruguay. *Anales del Museo Nacional de Historia Natural de Montevideo* 1935; **4**: 1–96.

15. **Acosta y Lara E**. Quirópteros del Uruguay. *Comunicaciones Zoológicas del Museo de Historia Natural de Montevideo* 1950; **3**: 1–73.

16. **Acosta y Lara E**. Observaciones sobre una colonia de Desmodus rotundus (E. Geoffroy) en el Cerro Salamanca, Dpto. de Maldonado. *Comunicaciones Zoológicas del Museo de Historia Natural de Montevideo* 1959; **4**: 1–4.

17. **Langguth A, Achaval F**. Notas ecológicas sobre el vampiro Desmodus rotundus rotundus (Geoffroy) en el Uruguay. *Neotropica* 1972; **18**: 45–53.

18. **González EM, Martínez-Lanfranco JA**. *Mamíferos de Uruguay. Guía de campo e introducción a su estudio y conservación*. Montevideo: Vida Silvestre – Museo Nacional de Historia Natural, Ediciones de la Banda Oriental, 2010.

19. **CONAHSA**. *Acta 121 Comisión Nacional Honoraria de Salud Animal*. 2007, p. 1–11.

20. **CONAHSA**. *Acta 128 Comisión Nacional Honoraria de Salud Animal*. 2008, p. 1–9.

21. **CONAHSA**. *Acta 130 Comisión Nacional Honoraria de Salud Animal*. 2008, p. 1–13.

22. **CONAHSA**. *Acta 205 Comisión Nacional Honoraria de Salud Animal*. 2014, p. 1–8.

23. **Curbelo C, Bracco R**. La construcción del espacio misionero y la toponimia en territorio uruguayo. *II Congreso Nacional de Arqueología Histórica Argentina 2005* 2008; : 1–10.

24. **Azarola Gil LE**. Hernandarias de Saavedra y la primera exploración del Uruguay. *Boletín de la Real Academia de Historia* 1933; **102**: 158–182.

25. **Coni EA**. Historia de las vaquerías de Río de la Plata (1555-1750). *Boletín de la Real Academia de la Historia* 1930; **96**: 262–357.

26. **Soiza Larrosa A**. Un médico inglés en el Río de la Plata antes de la fundación de Montevideo. *Revista del Instituto Histórico y Geográfico del Uruguay* 2010; **23**: 9–50.

27. **Toller W**. *The history of a voyage to the River of Plate & Buenos Aires from England*. London, 1715.

28. **Barrios Pintos A**. *400 años de historia de la ganadería de Uruguay*. 2da Edició. Montevideo: Ediciones Cruz del Sur, 2011.

29. **Perez Castellano JM**. *Selección de Escritos. Crónicas históricas 1787-1814*. García Capurro F et al., eds. Montevideo: Ministerio de Educación y Cultura, 1968.

30. **Mawe J**. *Travels in the interior of Brazil, particulary in the gold and diamond districts of that country, by authority of the Prince Regent of Portugal: including a voyage to the Rio de le Plata and an historical sketch of the revolution of Buenos Ayres*. London: Longman, Hurst, Rees Orme and Brown, 1812.

31. **Christison D**. A journey to central Uruguay. *Proceedings of the Royal Geographical Society* 1880; **2**: 663–689.

32. **Bertino M, Tajam H**. *La ganaderia en el uruguay 1911 – 1943*. *Documentos de Trabajo*. Montevideo, 2000.

33. **OPYPA Oficina de Programación y Política Agropecuaria**. *Anuario 2016*. Montevideo, 2016.

34. **Dirección de Estadísticas Agropecuarias**. *Censo General Agropecuario. Resultados definitivos*. Montevideo, 2011.

35. **Vaz Ferreira R**. *Estudio preliminar*. In: Narancio EM, Vaz Ferreira R, eds. *Viaje de William Toller a la Banda Oriental y Río de la Plata en 1715*. Montevideo: Instituto de Investigaciones Históricas y Laboratorio de Zoología, 1955, p. 1–5.

36. **Grubb P**. *Order Artiodactyla*. In: Wilson DE, Reeder DM, eds. *Mammal species of the world. A taxonomic and geographic reference*. 3rd ed. Baltimore: The Johns Hopkins University Press, 2005, p. 637–722.

37. **Mayer JJ, Wetzel RM**. Catagonus wagneri. *Mammalian Species* 1986; **259**: 1–5.

38. **Azara F de**. *Apuntamientos para la historia natural de los quadrúpedos del Paragüay y Rio de la Plata. Tomo primero*. Madrid: Imprenta de la Viuda de Ibarra, 1802.

39. **Kiltie RA, Terborgh JW**. Observations on the behavior of rain forest peccaries in Perú: Why do white-lipped peccaries form herds? *Zeitschrift für Tierpsychologie--Journal of Comparative Ethology* 1983; **62**: 241–255.

40. **Mayer JJ, Wetzel RM**. Tayassu pecari. *Mammalian Species* 1987; **293**: 1–7.

41. **Desbiez ALJ, *et al.*** Niche partitioning among white-lipped peccaries (Tayassu pecari), collared peccaries (Pecari tajacu), and feral pigs (Sus Scrofa). *Journal of Mammalogy* 2009; **90**: 119–128.

42. **Mones A, Klappenbach MA**. Un ilustrado aragonés en el Virrreinato del Río de la Plata: Félix de Azara (1742-1821) Estudios sobre su vida, su obra y su pensamiento. *Anales del Museo Nacional de Historia Natural de Montevideo* 1997; **9**: 1–232.

43. **Darwin C**. *Viaje de un naturalista alrededor del mundo*. Primera ed. Gil J, ed. Buenos Aires: Librería el Ateneo, 1945.

44. **Darwin C**. *Geographical introduction*. In: Darwin C, ed. *The zoology of the voyage of H.M.S. Beagle, under the command of Captain Fitzroy R.N. during the years 1832 to 1836*. London: Smith, Elder and Co., 1839, p. i–v.

45. **Aplin OV**. Field-notes on the mammals of Uruguay. *Proceedings of The Zoological Society of London* 1894; **1894**: 297–315.

46. **Weber M, Gonzalez S**. Latin American deer diversity and conservation: A review of status and distribution. *Ecoscience* 2003; **10**: 443–454.

47. **Aristimuño MP, González S, Barbanti Duarte JM**. Population structure and genetic variability of the gray-brocket deer (Mazama gouazoubira; Mammalia: Cervidae) in Uruguayan populations. *Deer Specialist Group Newsletter* 2015; **27**: 58–66.

48. **Black-Décima P, *et al.*** *Brown brocket deer Mazama gouazoubira (Fisher, 1814)*. In: Barbanti Duarte JM, González S, eds. *Neotropical Cervidology. Biology and medicine of Latin American deer*. Joboticabal and Gland: Funep - IUCN, 2010, p. 190–201.

49. **Delpietro HA, *et al.*** Observations of sylvatic rabies in Northern Argentina during outbreaks of paralytic cattle rabies transmitted by vampire bats (Desmodus rotundus). *Journal of wildlife diseases* 2009; **45**: 1169–1173.

50. **Pereira-Garbero R, *et al.*** Mamíferos invasores en Urugay, historia, perspectivas y consecuencias. *Revista Chilena de Historia Natural* 2013; **86**: 403–421.

51. **González EM, *et al.*** *Mamíferos*. In: Soutullo A, Clavijo C, Martínez-Lanfranco JA, eds. *Especies prioritarias para la conservación en Uruguay. Vertebrados, moluscos continentales y plantas vasculares*. 1st ed. Montevideo: Sistema Nacional de Áreas Protegidas, 2013, p. 175–207.

52. **Bobrowiec PED, Lemes MR, Gribel R**. Prey preference of the common vampire bat (Desmodus rotundus, Chiroptera) using molecular analysis. *Journal of Mammalogy* 2015; **96**: 54–63.

53. **Galetti M, *et al.*** Liquid lunch - vampire bats feed on invasive feral pigs and other ungulates. *Frontiers in Ecology and the Environment* 2016; **14**: 505–506.

54. **Hayes MA, Piaggio AJ**. Assessing the potential impacts of a changing climate on the distribution of a rabies virus vector. *PLoS ONE* 2018; **13**: e0192887.

55. **Lee DN, Papes M, Bussche RA Van Den**. Present and potential future distribution of common vampire bats in the americas and the associated risk to cattle. *PLoS ONE* 2012; **7**: e42466.

56. **González EM**. *Guía de campo de los mamíferos de Uruguay. Introducción al estudio de los mamíferos*. Montevideo: Vida Silvestre, 2001.

57. **Botto Nuñez G, *et al.*** The first artificial intelligence algorithm for identification of bat species in Uruguay. *Ecological Informatics* 2018; **46**: 97–102.
