# Supplementary material for: The emergence of vampire bat rabies in Uruguay within a historical context
Source: Epidemiol Infect. 2019 Apr 22;147:e180. doi: 10.1017/S0950268819000682 (PMC6518465; doi:10.1017/S0950268819000682)
Supplement: Supplementary file 1 [file S0950268819000682sup001.zip › S0950268819000682sup001/tables_supmat_altver.docx]

**Epidemiology and Infection**

**The emergence of vampire bat rabies in Uruguay within a historical context**

Germán Botto Nuñez, Daniel J. Becker, & Raina K. Plowright

Table S1. Historical records on cattle abundance, and wildlife and vampire bat distribution in Uruguay since the 1500s

| **Year** | **Cattle** | **Wildlife** | **Vampire bats** | **References** |
| --- | --- | --- | --- | --- |
| 1552 / 1568 | Introduction of cattle from Paraguay and Bolivia for the foundation of Santa Fe. Later that cattle were introduced into Uruguayan territory |  |  | [1,2] |
| 1611 / 1617 | First and second introduction of cattle by Hernandarias |  |  | [3] |
| 1627 | Estimation of a minimum of 100.000 cattle in Uruguayan territory |  |  | [2] |
| 1702 – 1714 | 174.000 leather pieces exported from Buenos Aires (including Uruguayan ones) |  |  | [4] |
| 1710 | Start of systematic exploitation of cattle in Uruguay |  |  | [2] |
| 1715 | Reports of very abundant cattle in the coast by Europeans visitors | Deer (probably pampa’s deer) in large herds. Capibaras and peccaries also in large groups near the coast. |  | [4,5] |
| 1723 – 1729 | 480.000 leather pieces exported from Colonia |  |  | [4] |
| 1777 | 364.000 leather pieces exported from Montevideo (plus and unknown number of smuggled pieces) |  |  | [6] |
| 1780 | 480.000 cattle in Montevideo’s jurisdiction, by census |  |  | [6] |
| 1781 | Convoy from Montevideo exported 432.000 leather to Europe. 6 mail frigates also carrying leather left from Montevideo. |  |  | [7] |
| 1787 | 321.450 leather pieces exported from Montevideo |  |  | [7] |
| 1792 – 1793 | 1.6 million leather pieces exported from Montevideo |  |  | [6] |
| 1800 | Leather export paralyzed in the country |  |  |  |
| 1806 | English geologist mentions several ranches within 145 km east from Montevideo, ranging from 60.000 to 200.000 cattle each. | Large herds of pampa’s deer. Peccaries also present in the south-central area of the country |  | [8] |
| 1821 | 2.6 million cattle just in possession by Brazilians in southern Uruguay |  |  | [6] |
| 1833 |  | Pampa’s deer and capybaras are very abundant |  | [9] |
| 1867 | English naturalist describes a standard ranch in central Uruguay and calculates a 64 cattle per square km density | Pampa’s deer not uncommon but in lower numbers. Swamp deer absent at that time but was previously abundant. Jaguar and great anteaters were also absent by that time. Puma was still present, but already rare |  | [10] |
| 1887 | 12 million cattle estimated in the country (5.2 million in possession by Brazilians in the whole country) |  |  | [6] |
| 1892 |  | Pampa’s deer almost exterminated in some jurisdictions, but still abundant in the center of the country. Swamp deer only present in riparian forests of the Uruguay river. |  | [11] |
| 1894 |  | Figueira considers pampa’s deer still abundant in the southeastern region. Swamp deer present only in the north | Only five species of bats recorded for Uruguay. *Desmodus* is not mentioned. | [12] |
| 1908 | 8.2 million cattle, 21.5 million sheep. Official census data |  |  | [13] |
| 1916 | 7.8 million cattle, 11.5 million sheep. Official census data |  |  |  |
| 1920 - 1930 |  | Introduction of Axis deer, Dama deer, and wild boars in the southwest |  | [14] |
| 1924 | 8.4 million cattle, 14.4 million sheep. Official census data |  |  |  |
| 1926 |  |  | Sanborn visited Uruguay, at the Arequita cave he found only *Myotis levis*, but not *D. rotundus* | [15] |
| 1930 | 7.1 million cattle, 20.5 million sheep. Official census data |  |  |  |
| 1933 |  |  | First *Desmodus* collected in northern Uruguay | [16] |
| 1935 |  |  | Devincenzi cites the species based on the 1933’s individual but consider it as a rare species in the country | [16] |
| 1937 | 8.3 million cattle, 17.9 million sheep. Official census data |  |  |  |
| 1943 | 6.3 million cattle, 15.4 million sheep. Official census data |  |  |  |
| 1946 | 6.8 million cattle, 19.6 million sheep. Official census data |  |  |  |
| 1950 |  |  | Following field work in northeastern Uruguay, Acosta y Lara considers the species to be “unexpectedly common”, with large (hundreds of individuals) colonies. |  |
| 1959 |  |  | Acosta y Lara publishes a list of 10 localities for the species in the north and eastern regions of the country | [17] |
| 1969 |  |  | First record of vampire bats in Arequita cave. This is the only cave where the species was recorded after the being visited several times by mammalogists. | [18] |
| 1972 |  |  | *Desmodus rotundus* considered to be abundant and widely distributed in the country. | [18] |
| 1976 - 1978 |  | 9 populations of pampas deer with an estimated total of less than 1.000 individuals |  |  |
| 1980s |  | Introduction of Asian water buffalo |  |  |
| 1982 |  | Wild boars declared agricultural pest |  |  |
| 1998 |  | Estimated 1.000 pampa’s deer, restricted to 2 populations |  | [19] |
| 2003 |  | Estimated 800 pampa’s deer, restricted to 2 populations |  | [20] |
| 2011 | 10.7 million cattle, 7.3 million sheep (Official Census) |  |  | [21] |
| 2013 |  | Wild boars abundant and widely distributed. Water buffalos restricted to two small populations. Dama deer restricted to the center of the country and dependent on conservation measures, because of hunting. Axis deer widely distributed in the country |  | [14] |
| 2013 – 2018 |  |  | The species is abundant and widely distributed. It appears very often in acoustic surveys regardless of habitat or location | Pers. Obs. Unpub. data. |
| 2016 | Estimated 12.1 million cattle. Estimated 2.5 million individuals removed annually from stock |  |  | [21] |

References.

1. **Curbelo C, Bracco R**. La construcción del espacio misionero y la toponimia en territorio uruguayo. *II Congreso Nacional de Arqueología Histórica Argentina 2005* 2008; : 1–10.

2. **Coni EA**. Historia de las vaquerías de Río de la Plata (1555-1750). *Boletín de la Real Academia de la Historia* 1930; **96**: 262–357.

3. **Azarola Gil LE**. Hernandarias de Saavedra y la primera exploración del Uruguay. *Boletín de la Real Academia de Historia* 1933; **102**: 158–182.

4. **Soiza Larrosa A**. Un médico inglés en el Río de la Plata antes de la fundación de Montevideo. *Revista del Instituto Histórico y Geográfico del Uruguay* 2010; **23**: 9–50.

5. **Toller W**. *The history of a voyage to the River of Plate & Buenos Aires from England*. London, 1715.

6. **Barrios Pintos A**. *400 años de historia de la ganadería de Uruguay*. 2da Edició. Montevideo: Ediciones Cruz del Sur, 2011.

7. **Perez Castellano JM**. *Selección de Escritos. Crónicas históricas 1787-1814*. García Capurro F et al., eds. Montevideo: Ministerio de Educación y Cultura, 1968.

8. **Mawe J**. *Travels in the interior of Brazil, particulary in the gold and diamond districts of that country, by authority of the Prince Regent of Portugal: including a voyage to the Rio de le Plata and an historical sketch of the revolution of Buenos Ayres*. London: Longman, Hurst, Rees Orme and Brown, 1812.

9. **Darwin C**. *Viaje de un naturalista alrededor del mundo*. Primera ed. Gil J, ed. Buenos Aires: Librería el Ateneo, 1945.

10. **Christison D**. A journey to central Uruguay. *Proceedings of the Royal Geographical Society* 1880; **2**: 663–689.

11. **Aplin OV**. Field-notes on the mammals of Uruguay. *Proceedings of The Zoological Society of London* 1894; **1894**: 297–315.

12. **Figueira JH**. Enumeración de mamíferos. Contribución al conocimiento de la fauna uruguaya. *Anales del Museo Nacional de Historia Natural de Montevideo* 1894; **2**: 187–217.

13. **Bertino M, Tajam H**. *La ganaderia en el uruguay 1911 – 1943*. *Documentos de Trabajo*. Montevideo, 2000.

14. **Pereira-Garbero R, *et al.*** Mamíferos invasores en Urugay, historia, perspectivas y consecuencias. *Revista Chilena de Historia Natural* 2013; **86**: 403–421.

15. **Sanborn CC**. The land mammals of Uruguay. *Field Museum of Natural History Zoological Series* 1929; **17**: 147–165.

16. **Devincenzi GJ**. Mamíferos del Uruguay. *Anales del Museo Nacional de Historia Natural de Montevideo* 1935; **4**: 1–96.

17. **Acosta y Lara E**. Observaciones sobre una colonia de Desmodus rotundus (E. Geoffroy) en el Cerro Salamanca, Dpto. de Maldonado. *Comunicaciones Zoológicas del Museo de Historia Natural de Montevideo* 1959; **4**: 1–4.

18. **Langguth A, Achaval F**. Notas ecológicas sobre el vampiro Desmodus rotundus rotundus (Geoffroy) en el Uruguay. *Neotropica* 1972; **18**: 45–53.

19. **González S, *et al.*** Conservation genetics of the endangered Pampas deer (Ozotoceros bezoarticu). *Molecular ecology* 1998; **7**: 47–56.

20. **Weber M, Gonzalez S**. Latin American deer diversity and conservation: A review of status and distribution. *Ecoscience* 2003; **10**: 443–454.

21. **OPYPA Oficina de Programación y Política Agropecuaria**. *Anuario 2016*. Montevideo, 2016.
