# Supplementary figures and images for: The emergence of vampire bat rabies in Uruguay within a historical context
Source: Epidemiol Infect. 2019 Apr 22;147:e180. doi: 10.1017/S0950268819000682 (PMC6518465; doi:10.1017/S0950268819000682)

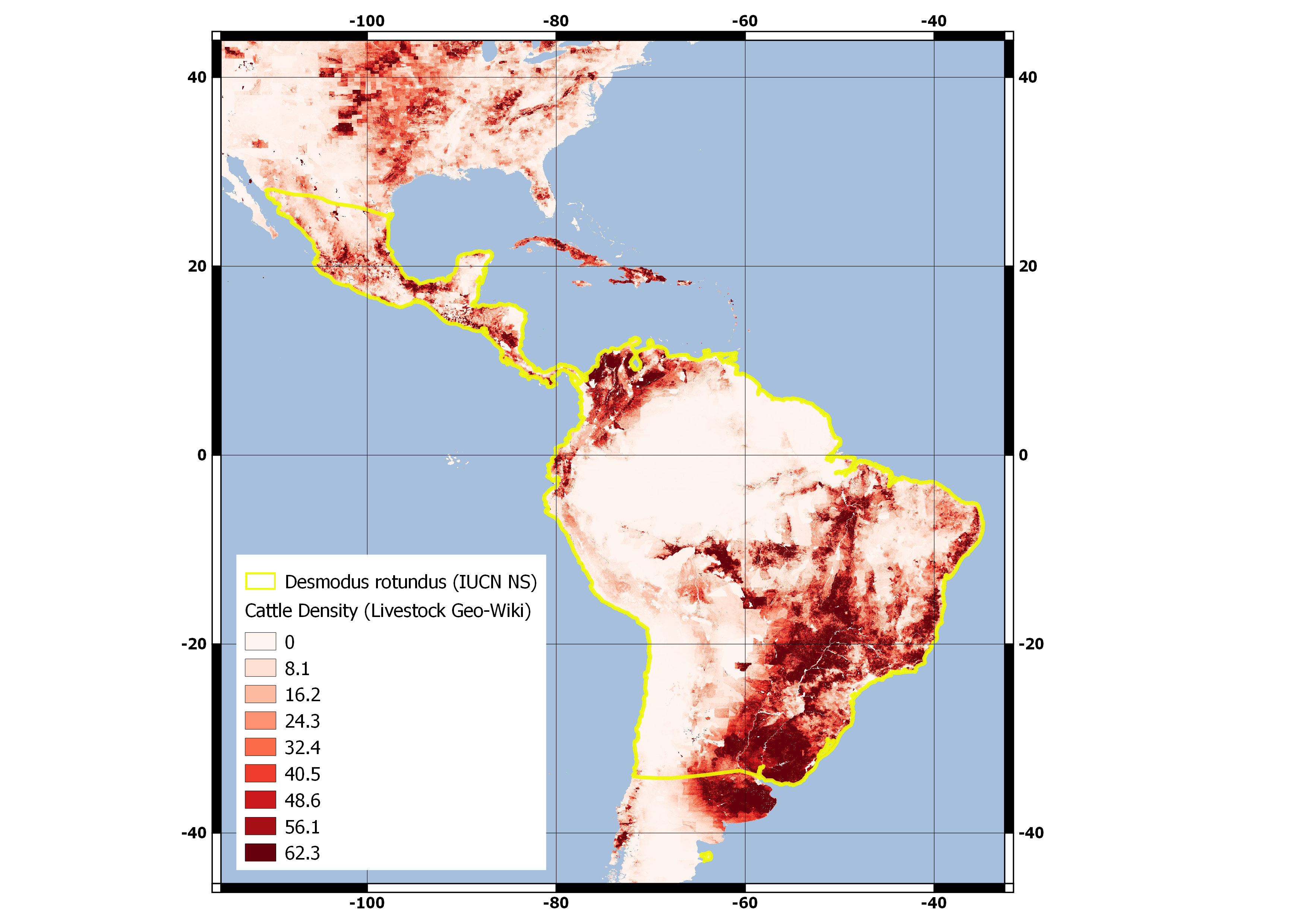

Supplement: Supplementary file 1 [file S0950268819000682sup001.zip › S0950268819000682sup001/Botto_FIGS1.png]

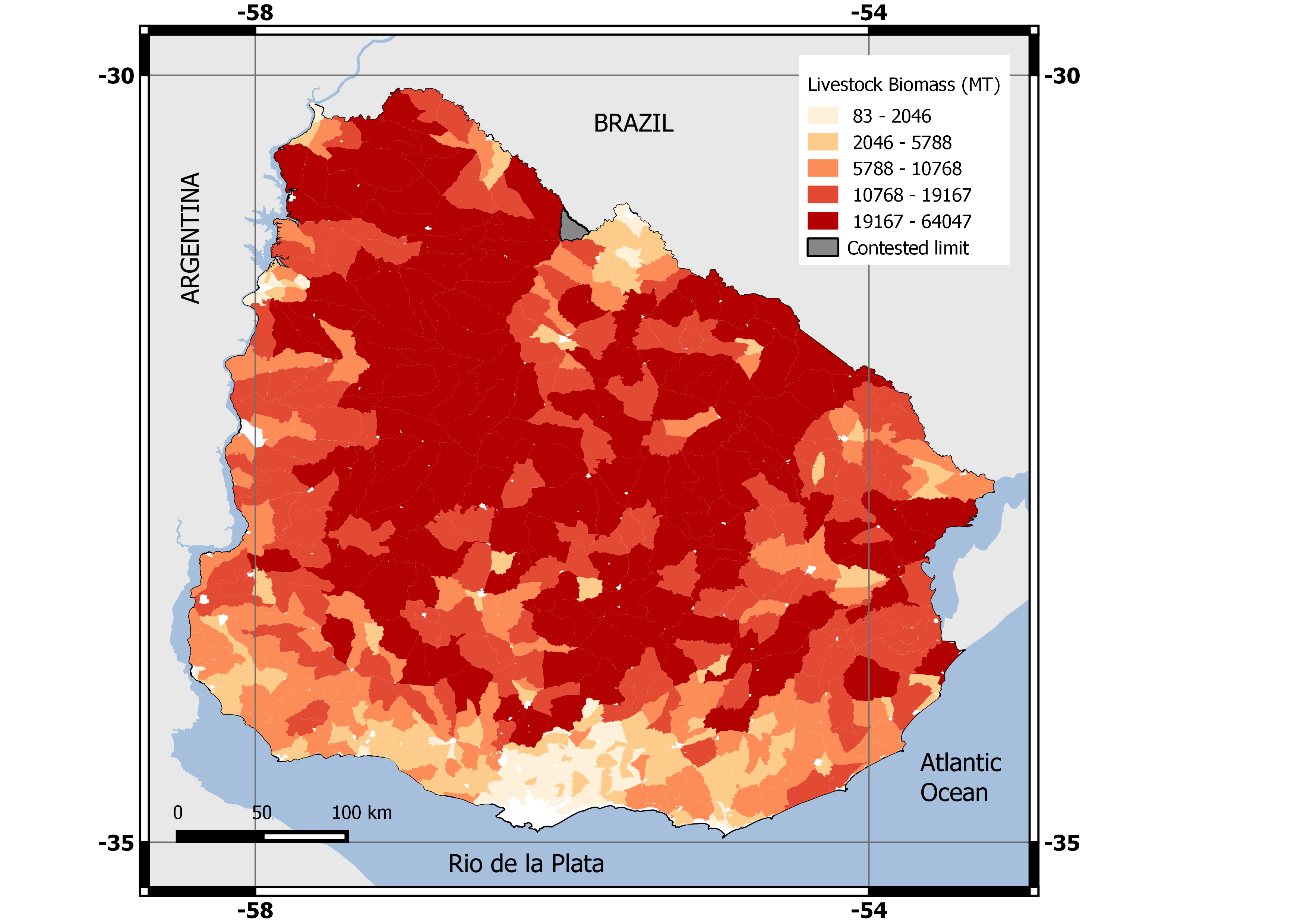

Supplement: Supplementary file 1 [file S0950268819000682sup001.zip › S0950268819000682sup001/Botto_FIGS2.png]
